# Targeting BCL2 in Waldenström macroglobulinemia: from biology to treatment management

**DOI:** 10.3389/fonc.2025.1564869

**Published:** 2025-04-22

**Authors:** Eleni Kalafati, Efstathios Kastritis, Tina Bagratuni

**Affiliations:** Department of Clinical Therapeutics, National and Kapodistrian University of Athens, School of Medicine, Athens, Greece

**Keywords:** BCL2, Waldenstöm’s macroglobulinemia, venetoclax, biology, inhibitor

## Abstract

Despite recent advances in the treatment of Waldenström macroglobulenimia (WM), including the development of Bruton tyrosine kinase inhibitors (BTKis), the disease remains incurable highlighting the urgent need for new treatments. The overexpression of *BCL2* in WM cells promotes cell survival by resisting apoptosis and contributes to resistance to chemotherapy and targeted therapies. Concurrently, Bcl2 proteins that are encoded by oncogenes supporting cell survival are frequently upregulated in WM, even in the presence of DNA-damaging agents, and hence have emerged as an alternative therapeutic target. Venetoclax serves as a novel orally administered small agent that targets Bcl-2 protein by acting as a *BCL2* homology domain 3 (BH3) mimetic and has shown promising results in WM patients, including those previously treated with BTKis. Furthermore, venetoclax, in combination with standard WM regimens, has shown enhanced activity, but further studies are required to elucidate the mechanism of its synergistic action and identify the patients who can benefit from the combined therapy. New BCL2 inhibitors are in advanced stages of clinical development and may offer additional options. The present review will focus on the current knowledge we have on BCL2 inhibitors in WM, the input of these compounds “from bench to bedside,” and their utility in managing relapsed/refractory WM patients.

## Introduction

Increased BCL2 expression is considered an important biomarker in the evaluation of aggressive disease and often associated with poor prognosis in Waldenström macroglobulenimia (WM) (1, 2). Given its critical role in cell survival, Bcl-2 has emerged as a therapeutic target in WM where the BCL2 inhibitor venetoclax targets the Bcl-2 protein by acting as a BCL-2 homology domain 3 (BH3) mimetic. The drug has been approved in the USA/EU for administration as a monotherapy or in combination with other regimens in chronic lymphocytic leukemia (CLL). It is in phase I/II trials for acute myeloid leukemia (AML), in phase III clinical trials for multiple myeloma (MM), and in phase I/II clinical studies for several B-cell malignancies, including WM (3, 4). Recent studies have demonstrated the safety and efficacy of venetoclax in patients with WM, including those previously treated with Bruton tyrosine kinase inhibitors (BTKi) (5, 6). Interestingly, WM cells lacking *BTK* or *CXCR4* mutations acquire resistance to BTKis through the upregulation of BCL2 and AKT resulting in vulnerability toward venetoclax administration. In the present review, we will summarize the current knowledge regarding the role of BCL2 in WM as well as the use of venetoclax for the treatment management of these patients.

## The BCL2 family

The BCL2 (B-cell lymphoma 2) protein family consists of key regulators of pro-apoptotic and anti-apoptotic proteins both critical for apoptotic cell death and maintenance of cellular homeostasis. Dysregulation of BCL2 family members is frequently linked to the development of cancer and other pathological conditions. The BCL2 family proteins are classified based on their function and the existence of retained Bcl-2 homology (BH) domains into anti-apoptotic, pro-apoptotic effector, and pro-apoptotic BH3-only proteins (7). Anti-apoptotic proteins, such as BCL2 (B-cell lymphoma 2), BCL-XL (BCL2-like 1), BCL-W (BCL2-like 2), MCL1 (myeloid cell leukemia 1), and BFL-1/A1 (BCL2-related protein A1) promote cell survival by inhibiting pro-apoptotic effectors. BCL2 family pro-apoptotic effector proteins, such as BAX (BCL2-associated X protein) and BAK (BCL2-antagonist/killer 1), are directly involved in permeabilizing the mitochondrial outer membrane to trigger apoptosis. Proteins, like BAD (BCL2-associated agonist of cell death), BID (BH3 interacting-domain death agonist), BIM (BCL2-like 11), PUMA (p53-upregulated modulator of apoptosis), and NOXA belong to the pro-apoptotic BH3-only proteins and act as initiators of apoptosis by binding and neutralizing anti-apoptotic BCL2 proteins or by activating effectors like BAX and BAK (8, 9). Anti-apoptotic members prevent cell death by sequestering pro-apoptotic proteins that induce mitochondrial outer membrane permeabilization (MOMP) leading to cytochrome c release and activation of caspases (10). Released cytochrome c binds to Apaf-1 (apoptotic protease-activating factor-1) in the cytoplasm forming the apoptosome. Subsequently, the apoptosome activates caspase-9, which in turn activates executioner caspases, such as caspase-3 and caspase-7, which degrade cellular components leading to apoptosis. By sequestering these BH3-only proteins, BCL-2 indirectly inhibits the activation of BAX and BAK, which are required for MOMP (11). Briefly, the canonical BCL-2 pathway is governed by interactions between BCL-2 and BCL-XL, which bind to BAK and BAX through their BH3-binding grooves preventing their activation and suppressing apoptosis. This interaction is mediated by BH3 domains, short α-helical motifs present in pro-apoptotic proteins that engage the hydrophobic binding pocket of BCL-2 and BCL-XL. Undoubtedly, the balance between these interactions ultimately determines cell fate (11, 12). In tumorigenesis, overexpression of anti-apoptotic proteins (e.g., BCL2) contributes to cancer progression by inhibiting cell death (12), where BCL-2 antagonizes BH3-only proteins (e.g., BID, BIM, BAD, PUMA, and NOXA), which act as upstream activators of apoptosis. It has also been shown that high BCL-2 levels are associated with resistance to chemotherapy and radiation (13, 14). Overall, the pro- and anti-apoptotic proteins create a network of interactions, which ultimately leads to cell fate decision.

BCL2 family proteins are critical in the development, activation, and survival of lymphocytes impacting immune responses and autoimmunity. BCL-2 supports the survival of immature B cells during their development in the bone marrow but it also prevents premature apoptosis of mature B cells in the peripheral lymphoid organs enabling adequate immune response capacity (15, 16). Moreover, BCL-2 is vital for the survival of naïve T cells and helps regulate the contraction phase of the immune response by balancing apoptosis in effector T cells after antigen clearance (17). The overexpression of BCL-2 in memory B and T cells promotes resistance to apoptosis enabling these cells to persist for longer periods of time, a procedure essential for the immunological memory and rapid response upon re-exposure to the same pathogens (18). By regulating apoptosis, BCL-2 also contributes to the elimination of autoreactive lymphocytes during central tolerance for T and B cells in the thymus and bone marrow, respectively (19). The inability to reduce BCL-2 expression in autoreactive lymphocytes may prolong the survival of the aforementioned cells increasing the risk for autoimmune diseases (16, 20). Furthermore, BCL-2 activity enhances the survival of regulatory T cells (Tregs), which is critical in blocking exorbitant immune responses and inflammation (21–24). Hence, the role of BCL2 is critical during an immune response by contributing to the survival of activated immune cells, ensuring that excessive or redundant cells are removed once the pathogen is cleared while preventing excessive inflammation and tissue damage (24, 25).

## Pathophysiology and genomics of Waldenström macroglobulinemia

WM is a rare lymphoplasmacytic lymphoma characterized by bone marrow (BM) infiltration by small lymphoplasmacytic lymphoma (LPL) cells exhibiting plasma cell differentiation and a monoclonal IgM protein production (26, 27). It is now established that WM arises from a mature B cell that has undergone somatic hypermutation (SHM) but not heavy-chain class switch (28, 29). Although approximately 25% of patients are asymptomatic at the time of diagnosis, when the disease progresses to its symptomatic stage, patients usually develop symptoms of anemia, peripheral neuropathy, bleeding, hyperviscosity, enlarged spleen and/or liver, and other constitutional symptoms (30–32). It is considered a disease of the elderly, with a median age at diagnosis in the late 60s (6, 33, 34). Patients with an IgM serum protein of less than 3 g/dl and a BM infiltration of less than 10% but no symptoms are diagnosed with the pre-malignant phase of the disease known as IgM monoclonal gammopathy of undetermined significance (MGUS) (26, 35, 36). IgM MGUS accounts for approximately 10%–20% of all monoclonal gammopathies. The presence of disease-associated symptoms distinguishes the symptomatic from the asymptomatic/smoldering WM (27); the asymptomatic WM (AWM) is defined by the presence of either an IgM level more than 3 g/dl and/or more than 10% bone marrow clonal lymphoplasmacytic infiltration but no evidence of end-organ damage (26, 37). Patients with IgM-MGUS and AWM are at higher risk of developing symptomatic WM (38, 39), and this transition, which is estimated at a rate of 1.5% and 10% per year (39, 40), respectively, is probably a multi-step process leading to the progression from a benign condition to an overt neoplastic disease (41, 42). As opposed to WM patients, those with IgM-MGUS and AWM do not typically necessitate treatment.

Our deeper understanding of WM pathogenesis has greatly advanced with the discovery that in almost every patient with WM, the clone harbors the somatic mutation L265P in *MYD88*. The first study by Treon showed that 91% of WM patients and 50% of IgM-MGUS patients had the somatic *MYD88* mutation suggesting that this could be a critical event in the progression of IgM-MGUS to WM (43). However, although *MYD88* status is important for the differential diagnostic workup among other lymphoproliferative neoplasms (44–46), it is insufficient on its own to explain the malignant transformation of IgM-MGUS to WM. Furthermore, there is a small fraction of patients (3%–7%) who lack mutations in *MYD88*, and this group of patients may have an increased risk of disease transformation and shorter overall survival (47–49). The second most commonly mutated gene in WM is the chemokine receptor *CXCR4* where mutations have mostly been identified in the C-terminal domain and are observed in nearly 40% of patients with WM (45, 48, 50). *CXCR4* mutations almost always co-occur with MYD88 mutations, although some rare patients with WT *MYD88* can also express these mutations (48–51). Unlike *MYD88*, mutated *CXCR4* is subclonal, and its absence in most cases of IgM-MGUS patients compared to WM patients suggests that these mutations are acquired after *MYD88* (50).

Other genetic events observed in WM include mutations in *ARID1A*, *CD79B* seen in approximately 8%–15% of WM patients, while *TP53* mutations are quite uncommon (<10% of cases) but are associated with resistance to chemotherapy and shorter overall survival (52–55). Furthermore, copy number alterations are also seen in WM patients with the most frequent one being the deletion of 6q, which has shown to be associated with progression to the symptomatic disease (56, 57).

More than 15 years ago, a collaborative effort formulated the International Prognostic Scoring System for WM (IPSSWM) (58) that stratified patients into three risk groups with 5-year survival rates of 87% in the low-risk, 68% in the intermediate-risk, and 36% in the high-risk groups (59). The criteria used to stratify the abovementioned patients were based on the hemoglobin level, platelet count, B2M, and serum monoclonal IgM level. When therapy is required, the alkylator cyclophosphamide with dexamethasone (DRC), purine analog bendamustine (BR), rituximab (anti-CD20 monoclonal antibody), and the proteasome inhibitor (PI) bortezomib with dexamethasone and cyclophosphamide (B-DRC) are commonly utilized with enhanced treatment efficacy (60, 61). Furthermore, the application of covalent BTK inhibitors (cBTKi) has demonstrated successful targeted treatment options for frontline and relapsed WM patients (62–64).

## The role of BCL-2 in WM

In B-cell malignancies, BCL-2 dysregulation usually arises from chromosomal translocations, such as the *t (* 14, 18)(q32;q21) translocation in follicular lymphoma (FL) or focal deletion of chromosome 13 (*del*[13q14]) in CLL, which result to lack of the negative regulatory miRNA-15a/16-1 of BCL-2 (65–67). Specifically in WM, given the fact that MYD88 and CXCR4 molecular pathways induce the downstream activation of NF-κB, which in turn induces Bcl-x_L_, WM cells are probably dependent on the anti-apoptotic BCL-x_L_ for survival. Gaudette et al. showed that the differentiating B cells become dependent on BCL-x_L_ expression for their survival, especially in cases where there is a loss of MCL-1 and BCL-2 and induction of BIM expression suggesting that plasma cell differentiation proceeds through a BCL-x_L_-dependent intermediate stage (68). The same group showed that WM cells express low levels of pro-apoptotic Bcl-2 family members, which reduce their sensitivity to apoptosis-inducing agents; however, they suggest that mitochondrial priming can be initiated by antagonism of miR-155 or cleavage of BID, which is expressed in WM (69). On the other hand, disturbance of the mitochondria-mediated apoptosis pathway by the upregulation of the pro-apoptotic genes *BCL2* and *MCL-1* has been observed in more than 80% of myeloma and WM cases and is linked to a more aggressive clinical course (70). Specifically, *BCL2* expression was correlated with resistance to the proteasome inhibitor bortezomib, while the inhibition of Bcl-2 protein increased the cytotoxicity of lenalidomide and dexamethasone therapy. Furthermore, BCL2 upregulation and activation of AKT signaling has been also associated with resistance to the BTKi ibrutinib, in WM cells harboring BTK^C481S^ or CXCR4^WHIM-like^ mutations (71). This study demonstrated that the induction of ibrutinib resistance in WM cells can occur independently of the above mutations, and persistent pressure from ibrutinib appears to activate compensatory AKT molecular pathway as well as upregulation of Bcl-2 family proteins supporting cell survival. On the contrary, sensitivity to BTKi is directly correlated with the downregulation of Bcl-2 and the increased expression of caspase-9 (72). However, the most frequent somatic mutation in WM, MYD88^L265P^, caused the accumulation of self-reactive B cells *in vivo* only when apoptosis was challenged by increased Bcl-2 levels (73). Last, in WM, BCL-2 overexpression is not as prevalent as in other B-cell malignancies, such as follicular lymphoma (FL), where BCL-2 translocation (t[14;18]) that juxtaposes the BCL2 gene and immunoglobulin locus is a hallmark. However, despite the heterogeneity in BCL2 expression in samples from WM patients, BCL-2 still plays a role in cell survival, and BCL-2 inhibitors, like venetoclax, have shown efficacy in most WM cases (69, 74, 75).

## BCL2 inhibitors

**Figure 1:**
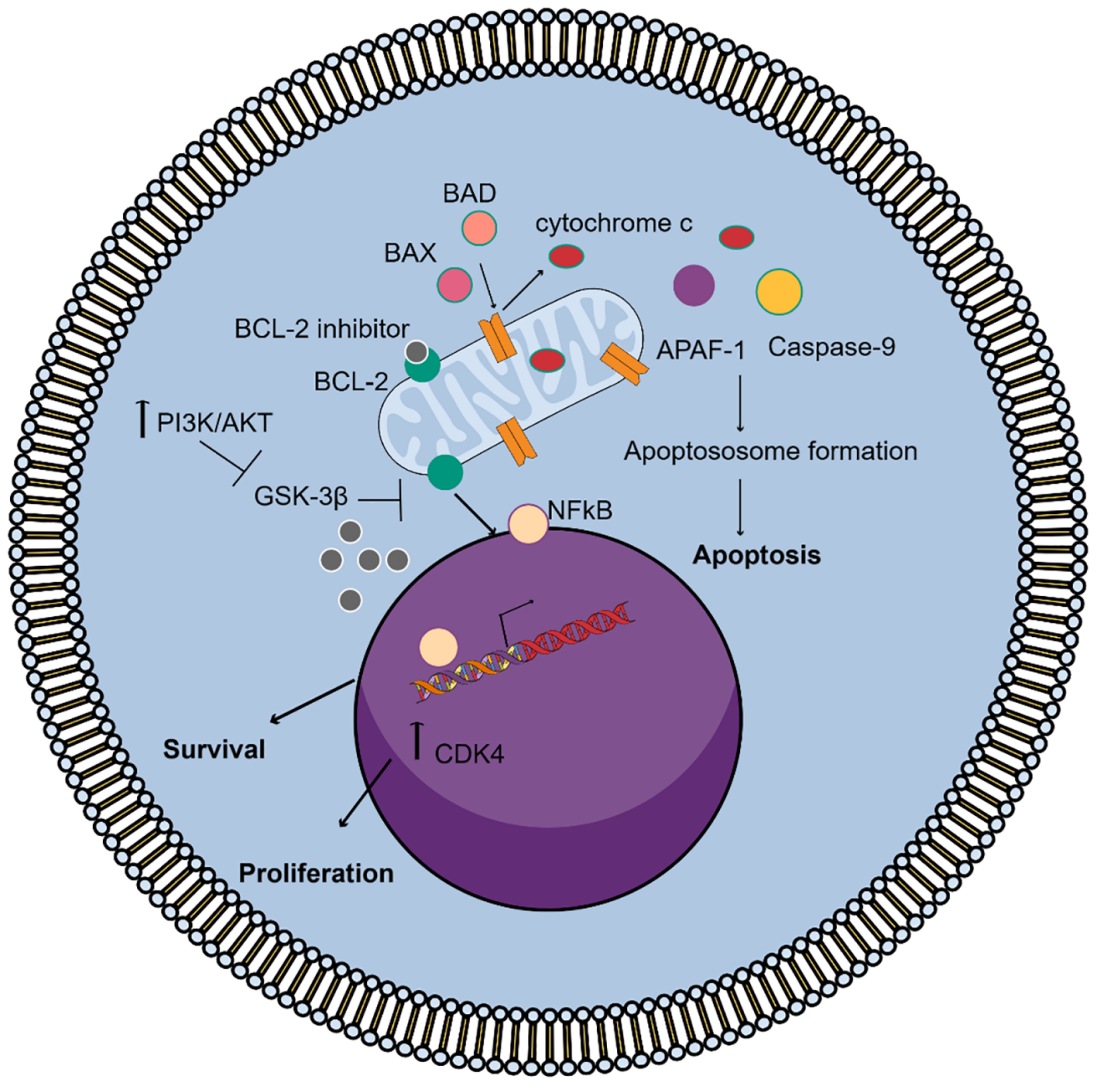
Mechanism of action of the BCL-2 inhibitors in WM. Under normal conditions, Bcl-2 binds and sequesters pro-apoptotic proteins, like BAX and BAK, preventing mitochondrial outer membrane permeabilization (MOMP) and blocking apoptosis. On the other hand, the activation of PI3K/AKT signaling increases Bcl-2 promoting cell survival and proliferation through NF-kB signaling and inhibits GSK3β activity, which regulates Bcl-2 family protein activity. BCL-2 inhibitors bind to Bcl-2 allowing BAX and BAK to oligomerize and form pores in the mitochondrial membrane. This leads to cytochrome c release, caspase activation, apoptosome formation, and irreversible apoptosis.

It is well established that many cancers, including WM, exploit the BCL2 pathway for survival making these proteins targets for cancer therapy. To address this cancer hallmark, significant efforts have been made to activate apoptotic cell death by inhibiting BCL-2 family proteins. It has been shown that cyclin-dependent kinase-4 (CDK4) protein expression was significantly downregulated in WM cells by DNA interference (DNAi), named PNT2258, targeting both intended (*BCL-2*) and unintended (*CDK4*) genes, while BCL-2 antisense therapy with G3139, an 18-base phosphorothioate oligonucleotide complementary to the first six codons of Bcl-2 mRNA, showed increased WM cell death and potential synergy with chemotherapeutic agents (76, 77). A set of intensely selective BCL-2 homology 3 (BH3) domain mimetics are in medical use and in current clinical trials for several subtypes of leukemia, multiple myeloma, and WM (78). The main mechanism of BCL-2 inhibitors’ action is depicted in **Figure 1**. Since the development of the first BH3 mimetic, ABT-737, there has been a rapid swift transition toward the use of small molecule inhibitors of anti-apoptotic BCL2 proteins (77, 78). To this end, the development of the anti-BCL2 inhibitor ABT-199, known as venetoclax, was key in the treatment management of numerous hematological malignancies (79, 80). Venetoclax is an orally administered, small-molecule apoptotic stimulant that targets BCL-2 proteins and has been approved in the USA and EU either as a single therapy or in combination with anti-CD20 monoclonal antibodies or with ibrutinib for patients with CLL, or in combination with a hypomethylating agent for patients with acute myeloid leukemia (AML) ineligible for intensive chemotherapy. In addition, venetoclax has been explored in the treatment of multiple myeloma, and a significant amount of data support its use in amyloid light-chain (AL) amyloidosis patients harboring the t (11, 14) translocation. Phase 2 studies have investigated the impact of venetoclax in WM, and larger studies are about to start to explore its combinations against standard WM regimens (81). Novel BCL-2 inhibitors, such as BGB-11417 (sonrotoclax), navitoclax, and lisaftoclax (APG-2575), are also tested in preclinical and clinical studies (82–84).

Preclinical studies showed that venetoclax enhanced the antitumor activity of KIN-8194, a dual inhibitor of hematopoietic cell kinase (HCK) and BTK in WM xenograft mice bearing wild-type or mutated *BTK* and *MYD88*-mutated cells and notably slowed tumor growth reducing cell survival (85). Even though bromodomain and extraterminal (BET) inhibitors reduce growth of WM cells, with a minimal effect on cell survival, the co-administration with venetoclax exhibits synergistic action augmenting the binding affinity of JQ1 (BET inhibitor) by 11.5 %, while JQ1 enhanced its binding affinity by 16.3% in comparison to the separate administration of each drug (5, 86).

## Clinical studies of BCL2 inhibitors in WM

At present, no BCL2 inhibitors are approved for the treatment of WM, while four clinical trials have investigated the efficacy of BCL2 inhibitors as single agents in WM patients (**Table 1**). The first was a phase I/II study initiated in 2003 (NCT00062244), at the Mayo clinic, in patients with WM. A total of 58 patients were enrolled between 2003 and 2007 received oblimersen sodium, an antisense oligonucleotide for the first six codons of the BCL2 open reading frame, which blocks BCL-2 production (87–89). In the WM cell line, the administration of oblimersen resulted in increased cytotoxicity upon exposure to rituximab, fludarabine, or dexamethasone. Oblimersen sodium was safely given to WM patients at a low dose level of 3 mg/kg per day. The most common toxicity detected was mainly hematologic, inducing thrombocytopenia and neutropenia, but the treatment was generally tolerable in elderly, heavily pretreated patients. The results of the phase I portion demonstrated a partial response in only one of nine enrolled patients (90). The clinical development of this agent has stopped.

**Table 1:** Completed and in-progress clinical trials of BCL2 inhibitors in WM.

Clinical trial	Phase	Therapeutic agent	Status	Start date	Completion date	Patients
NCT00062244	I/II	Oblimersen sodium	Completed	2003	2007	58 total 9 WM R/R
NCT01328626	I	Venetoclax	Completed	2011	2020	222 total 4 WM R/R
NCT02677324	II	Venetoclax	Completed	2016	2022	33 WM R/R
NCT04277637	Ia/Ib	Sonrotoclax	Ongoing	2020	2027	537 (estimated) 17 WM R/R
NCT05952037	II	Sonrotoclax	Ongoing	2023	2028	105 WM R/R (estimated)
NCT04273139	II	Venetoclax Ibrutinib	Early termination	2020	2028	45 WM R/R total
NCT04840602	II	Venetoclax Rituximab	Ongoing	2022	2028	92 (estimated) untreated WM/lymphoplasmacytic lymphoma
NCT05734495	II	Venetoclax Pirtobrutinib	Ongoing	2023	2033	42 (estimated) WM R/R

A phase I dose-escalation study by Davids and colleagues (NCT01328626) aimed to investigate the safety, pharmacokinetics, and effectiveness of venetoclax in patients with relapsed/refractory (R/R) non-Hodgkin lymphomas. The study was initiated in 2011 and included 106 patients of which only four were WM patients. Venetoclax was administrated once daily at a dose of 200–1,200 mg until disease progression or unexpected toxicity was observed. All four WM patients in this study achieved a partial response with a median time to first response of 2.6 months and a median duration of response at 25.3 months. None of the WM patients experienced tumor lysis syndrome (TLS), whereas hematologic side effects were less than 20%, with 49% patients experiencing nausea, 46% diarrhea, and 44% fatigue (91).

In 2016, a prospective phase II study of venetoclax monotherapy was initiated by Castillo and colleagues at the Dana Farber (NCT02677324). Thirty-two patients with relapsed or refractory WM were selected, with an average of one previous treatment line. Among them, 16 patients had been given earlier BTKi and seven were BTKi relapse. All patients harbored *MYD88^L265^* ^P^ mutation, while 17 patients carried *CXCR4^WHIM^* mutations. Venetoclax was orally administered on a daily basis at 200 mg for the first week, followed by 400 mg for the second week, and 800 mg for up to 2 years. Overall response rate (ORR) was 84%, and major response rate (MRR) was 81%, including 19% of patients who achieved VGPR, but no one attained a complete response (CR). Median progression-free survival (PFS) was 30 months. Six patients progressed within the first 24 months and 13 patients after completion of 2 years of venetoclax administration. Previous cBTKi administration was linked to lengthened response period. All 32 patients were alive at the time of data cut-off, with a 30-month overall survival (OS) rate of 100% (74, 92). Although the response rates were high, this study also showed that a fixed duration of venetoclax monotherapy may not be the optimal strategy to use this drug in WM.

A phase I trial of the Bcl-2 inhibitor BGB-11417 (sonrotoclax) in patients with mature B-cell malignancies was initiated in 2020, and the study is estimated to be completed in 2027 (NCT04277637). The purpose of this study is to determine the safety and tolerability of BGB-11417 as well as the determination of the maximum tolerated dose (88). After an average follow-up of 10.6 months, 4 (24%) out of 17 R/R WM patients have progressed, and 2 have discontinued due to adverse events. VGPR was 12%, MRR was 41%, and ORR was 76% (89). Another clinical trial initiated in 2023 evaluates the safety and efficiency of sonrotoclax in WM patients who are resistant to or cannot tolerate BKTi (NCT05952037). The study aims to recruit 105 participants in total, with completion expected by 2028. This study includes three cohorts. Cohort 1 refers to participants with R/R disease resistant to both BTKi and anti-CD20-containing chemotherapy. Cohort 2 includes patients with R/R disease resistant to anti-CD20-containing CIT with unresponsiveness and adverse reaction to BTKi. Cohort 3 includes patients with R/R disease resistant to BTKi and not appropriate for chemoimmunotherapy. Moreover, a fourth cohort will assess the co-administration of sonrotoclax and a non-covalent BTKi (cBTKi) zanubrutinib (89, 93). No preliminary data are yet available from this study.

## BCL2 inhibitors in combination with BTK inhibitors and other therapeutic factors

The combination of venetoclax with ibrutinib has been explored in CLL and associated with substantially high rates of complete responses with undetectable MRD. Thus, it has been postulated that the combination could also lead to very high rates of deep responses in patients with WM providing a fixed duration chemotherapy free treatment. A multicenter, single-arm prospective phase II study evaluated venetoclax in combination with ibrutinib in patients with previously untreated WM for a fixed duration of 2 years (NCT04273139). A total of 45 patients were recruited between 2020 and 2022, while patients bearing the *MYD88^WT^* genotype were exempted. Treatment was given every 28 days with the first cycle containing ibrutinib 420 mg, whereas venetoclax was administered once daily at 100 mg for the first week, 200 mg for the second week, and 400 mg for two more weeks. From cycles 3 to 24, participants received venetoclax at 400 mg and ibrutinib at 420 mg on a daily basis until unexpected side effects or disease progression were observed. The MRR was 96%, while the ORR was 100% with 53% PR, 42% attaining VGPR, and only 4% MR. The OS rate of 2 years was 96%, and PFS was 76%. However, three patients encountered ventricular arrhythmias, whereas two deaths and an individual grade-4 event were noted. The above patients had cardiac comorbidities, including a history of coronary artery disease, arrhythmia, hypertension, hypercholesterolemia, obesity, and/or diabetes. The clinical trial was subsequently discontinued after the existence of a grade-2 ventricular arrhythmia in a participant undergoing a precautionary cardiac stress test, while 9% of the participating patients exhibited ventricular arrhythmias. The investigators suggested the potential additive effects of BTK and BCL2 inhibitor combination in WM, including rapid responses, high rates of VGPR, and significant decrease in bone marrow burden, while a reduction from 60% to 5% at best response was observed. Although an unacceptably high incidence of ventricular events were observed, which could potentially be driven by undetected cardiac involvement of the primary amyloid, other cardiac paraprotein depositions, or an inherited increased risk of cardiac incidences occurred due to comorbidities (89, 94). Interestingly, such side effects were not observed in other B-cell malignancy trials when using ibrutinib and venetoclax combinations (95, 96).

Another randomized phase II study tests the combination of venetoclax and rituximab, an anti-CD20 monoclonal antibody versus ibrutinib and rituximab for the treatment of naïve lymphoplasmacytic lymphoma patients, including WM for a fixed duration of 2 years (NCT04840602). The clinical trial was initiated in 2022, with the purpose of encompassing a total of 92 patients, whereas its completion date is estimated to be in 2028 (97).

A phase II study evaluating venetoclax and pirtobrutinib (a noncovalent BTKi) in previously treated WM patients was started by Castillo and colleagues (NCT05734495). This study started in 2023 aiming to enroll 42 patients. The addition of venetoclax was started on cycle 2 at 100 mg/day for the first week, 200 mg/day for the second week, and 400 mg/day for the two following weeks, proceeded by pirtobrutinib at 200 mg/day and venetoclax at 400 mg/day given together for cycles 3–24. Between May 2023 and June 2024, 16 patients have been enrolled. VGPR was attained in nine patients, PR in five, and MR in two, while the rate of overall response was 100%. The median time to VGPR was 1.9 months (98). CXCR4 mutations and previous exposure to cBTKi did not appear to affect time mediated to VGPR. Last, two patients encountered disease progression in the second and fifth month, respectively (98, 99). **Table 1** summarizes the clinical trials.

## Resistance to BCL2 inhibitors in WM

Besides the fact that BCL2 inhibitors are promising candidates not only as single agents but also in combination treatment, drug resistance, tumor lysis syndrome, disease relapse, and cytopenias of clinical importance are rising issues associated with their current use (77, 78). The upregulation of BCL-2 and AKT pathways contribute significantly to drug resistance in WM. Specifically, resistance to therapies, like ibrutinib, can lead to an increased reliance on BCL-2 expression for malignant cell survival and inhibition of apoptosis rendering cells vulnerable to BCL-2 inhibitors, such as venetoclax, that target this pathway. Additionally, *CXCR4* mutations, often seen in WM patients, can mediate resistance through altered signaling that reduces the effectiveness of various drugs including BCL-2 inhibitors (70, 71, 100). BTK-mediated signaling was found to be significantly diminished, accompanied by alterations in PI3K/AKT pathway activity and genes/proteins involved in apoptosis regulation. Paulus et al. reported a reduction in WM cell viability when the AKT inhibitor MK22016 or venetoclax was combined with ibrutinib highlighting the potential for enhanced therapeutic effects. Treatment with ibrutinib appears to trigger compensatory activation of AKT signaling and a reorganization of BCL-2 family proteins facilitating cell survival. The improved antitumor efficacy observed with combination therapy supports the rationale for developing treatment strategies that integrate venetoclax and ibrutinib with PI3K/AKT inhibitors to combat drug-resistant WM (71). The mechanisms underlying resistance to BCL-2 inhibitors in WM remain poorly characterized. Hence, further research is essential to clarify the biological processes and pathways involved in this resistance, which could inform the development of more effective therapeutic strategies.

## Conclusion

Targeting Bcl-2 represents a novel and promising strategy for the treatment of WM. This is particularly interesting since, although patients undergoing different types of treatments demonstrate good response rates, there is an increasing rate of acquired resistance particularly to BTKi-based therapies. Hence, ongoing clinical trials using BCL2 inhibitors, either alone or in combination with BTKis or CD20 inhibitor, aim to induce deep and sustained response in WM patients. However, this approach should be taking into consideration the BCL2 dependence for survival during tumorigenesis allowing for the selection of patients that will most likely be benefited from the treatment of BCL2 inhibitors.
